# Explorative study to identify novel candidate genes related to oxaliplatin efficacy and toxicity using a DNA repair array

**DOI:** 10.1038/sj.bjc.6605134

**Published:** 2009-06-16

**Authors:** D M Kweekel, N F Antonini, J W R Nortier, C J A Punt, H Gelderblom, H-J Guchelaar

**Affiliations:** 1 Department of Clinical Pharmacy and Toxicology, Leiden University Medical Center, Leiden, The Netherlands; 2 Biometrics Department, Netherlands Cancer Institute (NKI), Amsterdam, The Netherlands; 3 Department of Clinical Oncology, Leiden University Medical Center, Leiden, The Netherlands; 4 Department of Oncology, Radboud University Nijmegen Medical Center, Nijmegen, The Netherlands

**Keywords:** SNP array, oxaliplatin, efficacy, toxicity, *ATM*, *ERCC5*

## Abstract

**Purpose:**

To identify new polymorphisms (single nucleotide polymorphisms, SNPs) in DNA repair pathways that are associated with efficacy and toxicity in patients receiving oxaliplatin and capecitabine for advanced colorectal cancer (ACC).

**Methods:**

We studied progression-free survival (PFS) in 91 ACC patients, of whom germ-line DNA was isolated and genotyped using an Asper Biotech array. Overall survival (OS) and toxicity were studied as secondary end points. A step-wise selection of SNPs was performed, involving univariate and multivariate log-rank tests and Cox regression analysis, with age and performance status as covariates.

**Results:**

A total of 81 SNPs in 46 genes on the array were selected for further analysis, based on genotyping success rates and minor allele frequencies. After step-wise selection, we found that homozygosity for the ataxia telangiectasia mutated gene (*ATM*) rs1801516 or excision repair cross-complementing gene (*ERCC5*) rs1047768 SNPs was associated with shorter PFS; however there were no significant associations (*P*>0.01) with OS or toxicity.

**Discussion:**

This is the first study describing the pathway gene approach for the selection of new candidate genes involved in oxaliplatin efficacy and toxicity. The results suggest that the *ATM* and *ERCC5* genes may be associated with oxaliplatin efficacy in ACC.

Oxaliplatin is a cytotoxic anti-tumour agent that is frequently used in advanced colorectal cancer (ACC; Punt, 2004). Its use results in platination of (tumour) DNA and the formation of platinum/DNA cross-links. Oxaliplatin adducts are lethal to cells, and may be removed from the DNA through a number of DNA repair pathways. Polymorphisms in the nucleotide excision repair (NER), base excision repair (BER) and mismatch repair (MMR) pathways were found to be associated with DNA repair after oxaliplatin exposure (reviewed by Kweekel *et al*, 2005).

The classical way of studying associations between drug effects and genetic variation is by the candidate gene method. This method involves the careful selection of single nucleotide polymorphisms (SNPs) based on the functionality of the genetic variant. However, replication of associations as described in the literature has not always been possible. For a large extent, this is due to the fact that most drug effects are considered as complex traits and are therefore not causally linked to one single gene or SNP. An alternative to the candidate gene method is the whole-genome approach (WGA). The main advantage of this method is that it is hypothesis free because it does not rely on current understanding of the pharmacokinetics and -dynamics of a drug. However, false-positive results that occur from multiple testing are a major concern in this type of research. The pathway gene method is a combination of both methods; instead of candidate genes, candidate pathways are selected based on the pharmacodynamic and pharmacokinetic behaviour of a drug. After selecting the appropriate pathways, SNPs in the corresponding genes are included in association analysis. Compared to the WGA, the pathway method involves a limited number of statistical association tests while at the same time allowing a broader part of the genome to be tested compared to the candidate gene method. The risk of false-positive findings is lower compared to WGA; also, because more enzymes in a pathway are studied, finding an association with the most relevant gene is more likely (Wang *et al*, 2007). Asper Biotech offers DNA repair chips that determine 100 SNPs in 56 genes, including SNPs in the homologous repair pathway, as well as SNPs in the BER, NER and MMR pathways. Moreover, it bears SNPs from several genes related to the control of cell-cycle and apoptosis pathways (including cyclin-dependent kinases p16 and p21, and tumour suppressor protein p53; http://www.asperbio.com/DNArepair.htm). These DNA repair chips are promising means of investigating associations with response or toxicity in patients receiving platinum derivatives, using the pathway gene approach.

The purpose of the current study was to perform an explorative association study of DNA repair pathway SNPs with progression-free survival (PFS) in ACC patients receiving with oxaliplatin combination therapy. Secondary end points were overall survival (OS) and toxicity.

## Materials and methods

### Patients

Germ-line DNA was obtained from peripheral blood of Caucasian patients with ACC who participated in the Dutch CAIRO trial. The inclusion criteria and the clinical results of this study have been published elsewhere (Koopman *et al*, 2006). Patients were included in the current side study if they were randomised to arm B (combinational chemotherapy, starting with first-line irinotecan plus capecitabine) and continued to second-line therapy, which consisted of oxaliplatin (130 mg m^−2^ on day 1) and capecitabine (1000 mg m^−2^, b.i.d. on days 1–14), every 3 weeks until progression or unacceptable toxicity. Dose reductions were performed for capecitabine in case of grades 2–4 toxicity as described previously (Van Cutsem *et al*, 2001). Oxaliplatin dose reductions of 25% were carried out in case of grade 4 haematological toxicity, febrile neutropenia and for persistent paresthesias (⩾14 days, grade 1 neurotoxicity) or temporary (7–14 days) painful paresthesias/functional impairment (grades ⩾2 neurotoxicity). Patients experiencing persistent grades ⩾2 neurotoxicity received a 50% oxaliplatin dose reduction. If haematological and non-haematological toxicities had not recovered to grade 1 before the next treatment cycle, oxaliplatin dose was delayed for a maximum of 2 weeks. If still not recovered by that time, patients went off-study. Prophylactic use of haematological growth factors was not permitted. The accrual period was from January 2003 to December 2004, and EDTA blood samples for genotyping were collected from December 2003 to March 2005 after a protocol amendment. The objective of this amendment was to perform genetic association studies regarding drug efficacy and toxicity. The study protocol and the amendment were approved by the local ethics committees. Written informed consent was obtained from all patients participating in the genetic association study before blood collection. Tumour evaluation was performed every three cycles according to RECIST criteria (Therasse *et al*, 2000) and toxicity was graded according to US National Cancer Institute Common Toxicity Criteria, version 2.0. The primary end point of this study was association of individual SNPs with PFS. Secondary end points were associations with OS and the incidence of overall worst grades 3–4 toxicity. Progression-free survival was calculated as the time from the start of second-line treatment with oxaliplatin to progression, death or loss to follow-up, whichever came first. Overall survival was also calculated as the time from the start of second-line oxaliplatin treatment to death or loss to follow-up. Progression-free survival was preferred as primary end point over OS, because it reflects oxaliplatin efficacy and is not potentially influenced by salvage therapies.

### Genotyping

Peripheral EDTA blood was stored at −20°C before isolation with the Magnapure LC (Roche Diagnostics, Almere, the Netherlands) according to the manufacturer's instructions. Asper Biotech (Tartu, Estonia) designed and performed a DNA repair chip (including 100 DNA repair SNPs in 55 genes) according to proprietary protocols. The chip contained SNPs of the following pathways: BER (*XRCC1*, *XRCC2*, *XRCC3*, *XRCC4*, *XRCC5*, *XRCC9*, *APEX*, *POLB*, *LIG4*, *MYH*, *OGG1*), NER (*ERCC1*, *ERCC2*, *ERCC4*, *ERCC5*, *LIG1*, *LIG3*, *RAD23B*, *XPA*, *XPC*), MMR (*MLH1*, *MSH2*, *MSH3*, *MSH6*, *PMS2*, *RECQL*), double-strand break repair and HR (homologous recombination: *BARD1*, *BRCA1*, *BRCA2*, *FANCD2*, *NBS1*, *RAD51*, *RAD52*, *RAD54B*, *PARP1*, *PARP4*), cell-cycle regulation (*ATM*, *CCND1*, *CCNH*, *CDKN1B*, *CDKN2B*, *CDK7*, *CHEK2*, *RAD9A*, *TP53*, *TP53BP1*, *TP53BP2*, *p21*, *CDKN2A*, *GRTH*, *GADD45A*) and other enzymes involved in DNA synthesis (*CDA*, *NT5E*, *PCNA*, *MGMT*).

As a quality control check, we compared the results of the Asper Biotech array with six SNPs that had previously been determined by our own laboratory in the same set of 91 DNA samples (Van der Straaten *et al*, 2006) and unpublished data (available on request). We found that, depending on the SNP, only 0.0–3.6% of the samples showed results that were discordant, which suggests good quality of the array and the procedures that were used in genotyping. All genotype data are available on request.

### Statistics

Patients randomised to the combination treatment who started second-line oxaliplatin/capecitabine combination therapy and had SNP assessment were eligible for the analysis (*N*=91). A SNP was considered evaluable for analysis if at least 90% of the patients were successfully genotyped, and if the minor allele frequency (MAF) was at least 5%.

Log-rank tests and Cox proportional hazards regression were performed to investigate the association with PFS/OS. Logistic regression analysis was performed to investigate the association with toxicity. If more than three patients were homozygous for the mutant allele, we tested both the separate genotypes individually and the combination of genotypes using the dominant or recessive model (carrier analysis). If three or less patients were homozygous mutant, we only compared the wild-type patients with the mutant allele carriers.

The purpose of this study was to explore the possible associations of the clinical end points of each SNP, either as a separate factor or combined. We used a two-step approach. In the first step, we performed the univariate analysis of PFS, OS and overall worst grades 3–4 toxicity for each SNP separately. Single nucleotide polymorphisms with a *P*-value of <0.01 were considered statistically significant and selected for the next step. In the second step, a covariate analysis with age and a performance status of 2 at the start of therapy (according to WHO guidelines) was performed for each SNP separately. On the basis of previously published results (Koopman *et al*, 2007), both a WHO performance status of 2 and abnormal serum lactate dehydrogenase (LDH) should ideally have been selected as covariates. However, LDH was underreported in most patients at the start of second-line treatment and therefore not used in the analysis. Age was selected as a covariate because it generally associates with survival. Finally, SNPs with a *P*-value of <0.01 were analysed in combination, in one multivariate model with the same covariates as in the previous step. This last analysis was performed for exploratory purposes only, to evaluate the relative importance of the individual factors.

Correction for multiple testing was carried out. However, a Bonferroni correction (based on the number of SNPs) would be far too conservative in this case, because it assumes that the SNPs investigated are all independent. Clearly, many of these SNPs are not only in linkage disequilibrium but also the genes studied are part of interacting pathways. Therefore, the only rational option to correct for multiple testing is to account for the number of pathways tested (in this case BER, NER, MMR, HR and cell-cycle regulation, *n*=5). One could argue that correcting for the number of pathways even is too conservative, because these particular pathways are not independent entities either (Curtis *et al*, 2005). Moreover, a strict Bonferroni correction would negatively affect the explorative nature of the study and would increase the chance for false-negative results. For these reasons, we decided to set *α* at 0.01. Throughout the paper, we present unadjusted *P*-values.

## Results

A total of 91 patients were eligible for the current analysis. DNA from these patients was genotyped using an SNP array with 100 SNPs on 55 genes. Two of these SNPs were excluded from analysis because of low genotyping success rates (<90%). Seventeen SNPs were excluded because of MAF <5%. The remaining 81 SNPs in 46 genes were used for analysis of PFS, OS and grades 3–4 toxicity. All patients had genotype call rates exceeding 90%.

### Step I: univariate analysis of oxaliplatin efficacy and toxicity

Table 1 shows the SNPs for which the univariate analysis of PFS and OS yielded log-rank *P*-values <0.05 (*P* for overall log rank). Among others, these include the genes *ATM* (ataxia telangiectasia mutated gene, two SNPs), *OGG1* (8-oxoguanine DNA glycosylase gene), *LIG4* (DNA ligase IV gene, two SNPs) and *GADD45A* (growth arrest and DNA-damage-inducible gene *α*). The *ATM*, *GADD45A* and *OGG1* SNPs show (a trend towards) association with both PFS and OS. On the basis of their *P*-values, we selected the *ATM* (rs1801516), *ERCC5* (rs1047768) and *GADD45A* (rs532446) SNPs for further analysis of PFS; the *LIG4* (rs1805388) and *BARD1* (rs2070093) SNPs were selected for further analysis of OS.

With regard to overall toxicity grades 3–4, the genes *O*-6-methylguanine-DNA methyltransferase (*MGMT AGT*, rs1803965 and rs12917) and ligase I (*LIG1*, rs3730849) showed *P*-values of 0.016, 0.023 and 0.031 respectively. *ERCC2* (rs238406) was the only SNP significantly associated with grades 3–4 toxicity (*P*=0.007). Carriers of the mutant allele showed a lower risk of developing grades 3–4 toxicity. As only the *ERCC2* SNP reached our criteria for significance, it was selected for further analysis.

### Step II: covariate analysis of oxaliplatin efficacy and toxicity

We continued the analysis of SNPs selected in the first step by individual multivariate analysis, using age and PS=2 as covariates. Genotype distributions of the selected SNPs are shown in Table 2. For OS, neither *LIG4* rs1805388 (*P*=0.017) nor *BARD1* rs2070093 (*P*=0.094) reached significance when corrected for the covariates. With regard to PFS however, we found that except for *GADD45A* (rs532446), the remaining *ATM* (rs1801516) and *ERCC5* (rs1047768) SNPs were significantly associated (Table 3). Homozygote carriers of the *ATM* variant allele have a 4.25 times increased risk (confidence interval, CI: 1.45–12.44) of progression on second-line combination chemotherapy of capecitabine plus oxaliplatin, compared to patients with the wild-type *ATM* (*P*=0.008). Patients homozygous for the *ERCC5* (excision repair cross-complementing gene, alternatively called *XPG*) variant allele also show an increased risk of progression compared to wild-type *ERCC5* patients (HR for homozygote variant patients: 2.85; CI: 1.42–5.71, *P*=0.003). Combined analysis of *GADD45A* (rs532446) with the covariates suggested no relevant association of this SNP with PFS (overall *P* for log-rank analysis: 0.216). To obtain information on the relative impact on PFS of the *ATM* rs1801516 and *ERCC5* rs1047768 SNPs, we performed a final multivariate analysis of both SNPs combined, corrected for performance status and age as covariates. We found that patients homozygous for the variant allele of *ATM* rs1801516 had a 3.2 times increased risk of progression (CI: 1.06–9.73, *P*=0.039), whereas this risk was 2.4 times increased in patients homozygous for the *ERCC5* rs1047768 variant allele (CI: 1.14–4.99, *P*=0.021).

With regard to toxicity, we found that the association of the *ERCC2* rs238406 SNP was diminished to *P*=0.018 after adjusting for the covariates. Patients carrying ⩾1 mutant *ERCC2* allele have a 0.28 times risk to experience grades 3–4 toxicity during treatment with oxaliplatin (CI: 0.10–0.81).

## Discussion

This is the first study describing the pathway gene approach for the selection of new candidate genes involved in oxaliplatin efficacy and toxicity. The results of this analysis suggest that *ATM* and *ERCC5* might be involved in the efficacy of oxaliplatin-containing chemotherapy for ACC. The current report, although explorative in nature, may serve as a basis for further studies in other patient populations.

The *ATM* gene product interacts with the *ERCC5* protein, and this complex prevents platinum-treatment-induced apoptosis (Colton *et al*, 2006). Two genetic variations in the *ATM* gene have already been studied in association with OS, including the intronic G60A (rs664143) and the T-77C (rs664677) SNP. Pancreatic cancer patients receiving gemcitabine and radiation with or without combined gemcitabine/cisplatin induction therapy have shorter OS if they carry the −77C variant (Li *et al*, 2006). A longer OS was reported in the same study population for patients who are homozygous for the 60A allele (Okazaki *et al*, 2008). The *ATM* SNP determined in the current study (rs1801516, or G5557A) has recently been shown to have an association with a reduced risk of breast cancer (Schrauder *et al*, 2008). This particular SNP is not in linkage with the *ATM* SNPs T-77C and G60A (*r*^2^=0.22, based on HapMap data phase II, March 2008). In the literature there are no data on the functionality of the Asp to Asn amino-acid substitution at this position. However, theoretically the substitution of the negatively charged Asp to neutral Asn may influence the ATM's interaction with other proteins. This may be a likely explanation, because the Asp amino acid at position 1853 appears to be highly conserved trough evolution (Schrauder *et al*, 2008).

The other variant, *ERCC5* (rs1047768, or T335C), has been studied in ACC and ovarian cancer patients. A longer OS and PFS were reported for homozygous wild-type ACC patients treated with oxaliplatin/capecitabine combination therapy (Monzo *et al*, 2007), and similarly, a shorter OS was reported for homozygous mutant patients with ovarian cancer, treated with platinum-based chemotherapy (Saldivar *et al*, 2007). Although the results of the current study confirm these previous findings, it remains uncertain why a synonymous or ‘silent’ SNP would have clinical impact as the amino-acid sequence is unaltered. One explanation may be that this genetic variant is in linkage with other SNPs that do influence the amino-acid sequence of ERCC5. Alternatively, this silent ERCC5 SNP may result in a different protein conformation and hence different substrate specificity or enzyme activity, as was shown for several silent SNPs such as SNPs in the gene encoding P-glycoprotein (Komar, 2007).

Some of the SNPs detected in the presently used array have already been studied before by other groups, and we will summarise their findings for comparison. Previous studies included the SNPs *ERCC1* rs11615 (C118T) and rs3212986 (C8092A), *ERCC2* rs13181 (Lys751Gln) and rs238406 (C499A) as well as *XRCC1* rs25487 (Arg399Gln). Besides the latter, none of these SNPs showed a (borderline) significant association with PFS or OS in the current univariate analysis. Various studies in colorectal cancer (Park *et al*, 2003; Stoehlmacher *et al*, 2004; Viguier *et al*, 2005; Ruzzo *et al*, 2007) and lung cancer patients (Su *et al*, 2007) suggest that the *ERCC1* 118CC genotype is associated with longer OS and a better response towards platinum agents. Another study reports no association of this SNP with OS (Zhou *et al*, 2004), but a significantly worse OS was found for patients carrying ⩾1 mutant allele of the other *ERCC1* SNP, C8092A. For this SNP, an association with platinum-induced gastrointestinal toxicity was found by others (Suk *et al*, 2005). Regarding *ERCC2*, most of the research has focused on Lys751Gln. Some authors report no association of this SNP with clinical outcome (Giachino *et al*, 2007; Monzo *et al*, 2007) or toxicity (Le Morvan *et al*, 2007), whereas others describe a better anti-tumour response (Park *et al*, 2001; Ruzzo *et al*, 2007), longer OS (Stoehlmacher *et al*, 2004) and increased haematological toxicity (Booton *et al*, 2006) in wild-type homozygotes. For the other *ERCC2* SNP, C499A, no association with OS or response has been reported (Park *et al*, 2001). Furthermore, the current study shows a trend towards longer OS in patients carrying ⩾1 mutant allele of *XRCC1* Arg399Gln, which is in contrast to other studies reporting no association (Giachino *et al*, 2007) or a shorter OS in patients with a mutant allele (Gurubhagavatula *et al*, 2004).

A general problem of studies investigating a large set of SNPs is the risk of false-positive findings; for this reason we set our threshold for significance at *α*=0.01. However, in this particular case, our major concern is false-negative results due to the relatively small sample size. This risk can only be minimised by using data from more patients, which, in turn, is not always feasible due to limited access to clinical data and patient samples. False-negative results due to low power can explain the inability to replicate the previously discussed associations found by other groups. However, the inability to replicate may also reflect differences in patient selection, publication bias, chance or low correlation of a marker with the outcome measure (Colhoun *et al*, 2003). Another concern of our study may be the low threshold that was set for the MAF (>5%). More specifically, it required that at least nine mutant alleles were genotyped for the given sample set. As a result, the power to detect associations with uncommon genetic variants was low. However, the commercial array included many SNPs with low MAFs (e.g. 17 SNPs were excluded because of MAF<5%). In our opinion, SNPs with low MAF should preferably not be included when developing an array.

In the palliative treatment of ACC, much progress has been made in recent years with the aid of pharmacogenetics. For instance, studies have revealed that patients with mutated *K-RAS* oncogene do not benefit from cetuximab treatment (Karapetis *et al*, 2008). Febrile neutropenia, one of the side effects of irinotecan, can be predicted by the uridine diphosphate glucuronosyl transferase (*UGT*)1A1 ^*^28 genotype (Kweekel *et al*, 2008). The results of the current study, although explorative in nature, need to be confirmed in a larger, independent cohort and may serve as a basis for new candidate SNP studies of genes located in the various DNA repair pathways.

## Figures and Tables

**Table 1:** Step I: SNPs with *P*<0.05 in univariate analysis of PFS, OS and grades 3–4 toxicity

		P*
*PFS*		
*ATM* rs1801516 (Asp1853Asn) Ataxia telangiectasia mutated gene	Regulator of tumour suppressor proteins p53 and BRCA1, checkpoint kinase CHK2, checkpoint proteins RAD17 and RAD9 and DNA repair protein NBS1.	**0.001**
		
*ERCC5* rs1047768 (T335C, His46His)	Involved in excision repair of UV-induced DNA damage.	**0.006**
*XPG*, excision repair cross-complementing (5) gene		
		
*GADD45A* rs532446 (T3812C) Growth arrest and DNA-damage-inducible gene, *α*	The protein product of this gene responds to environmental stresses by mediating activation of the p38/JNK pathway through MTK1/MEKK4 kinase.	**0.008**
		
*RAD51* rs1801320 (G287C, Arg 96Pro)	Involved in the homologous recombination and repair of DNA.	0.016
RecA homologue gene		
		
*ATM* rs609429 (IVS48+238 C>G) Ataxia telangiectasia mutated gene	Regulator of tumour suppressor proteins p53 and BRCA1, checkpoint kinase CHK2, checkpoint proteins RAD17 and RAD9, and DNA repair protein NBS1.	0.024
		
*OGG1* rs1052133 (Ser 326Cys) 8-Oxoguanine DNA glycosylase gene	Excision of 8-oxoguanine, a mutagenic base by-product that occurs as a result of exposure to reactive oxygen.	0.025
		
*BRCA2* rs15869 (105 bp 3′of STP A>C) Breast cancer 2, early onset gene	Involved in maintenance of genome stability, specifically the homologous recombination pathway for double-strand DNA repair.	0.033
		
		
*OS*		
*LIG4* rs1805388 (−176 C>T, Thr 9Ile) DNA ligase IV gene	DNA ligase that joins single-strand breaks through non-homologous end joining. This protein forms a complex with the X-ray repair cross-complementing protein 4 (XRCC4).	**0.008**
		
*BARD1* rs2070093 (His 506His)	Forms a complex with BRCA1 that may be an essential aspect of tumour suppression by BRCA1.	**0.009**
BRCA1-associated RING domain 1 gene		
		
*OGG1* rs1052133 (Ser 326Cys) 8-Oxoguanine DNA glycosylase gene	Excision of 8-oxoguanine, a mutagenic base by-product that occurs as a result of exposure to reactive oxygen.	0.015
		
*GADD45A* rs532446 (T3812C) Growth arrest and DNA-damage-inducible gene, *α*	The protein product of this gene responds to environmental stresses by mediating activation of the p38/JNK pathway through MTK1/MEKK4 kinase.	0.022
		
*ATM* rs609429 (IVS48+238 C>G) Ataxia telangiectasia mutated gene	Regulator of tumour suppressor proteins p53 and BRCA1, checkpoint kinase CHK2, checkpoint proteins RAD17 and RAD9, and DNA repair protein NBS1.	0.029
		
*LIG4* rs1805389 (−194 C>T) DNA ligase IV gene	DNA ligase that joins single-strand breaks through non-homologous end joining. This protein forms a complex with the X-ray repair cross-complementing protein 4 (XRCC4).	0.034
		
*MUTYH* rs3219489 (Gln 324His) MUT Y homologue	Encodes a DNA glycosylase involved in oxidative DNA damage repair; excises adenine bases that are inappropriately paired with, eg 8-oxo-7,8-dihydroguanine (major oxidative DNA damage).	0.049
		
*XRCC1* rs25487 (G1301A, Arg 399Gln) X-ray repair complementing defective repair (1)	Involved in the efficient repair of DNA single-strand breaks formed by exposure to ionizing radiation and alkylating agents.	0.049
		
		
*Toxicity*		
*ERCC2* rs238406 (C499A, Arg156Arg) XPD (excision repair cross-complementing rodent repair deficiency, complementation group 2)	The protein encoded by this gene is involved in transcription-coupled nucleotide excision repair of damaged DNA, and is an integral member of the basal transcription factor BTF2/TFIIH complex.	**0.007**
		
*MGMT* rs1803965 (C171T, Leu 53Leu)	DNA repair gene regulated by p53, confers resistance to alkylating agents	0.016
*O*-6-methylguanine-DNA methyltransferase		
		
*MGMT* rs12917 (C262T, Leu 84Phe)	DNA repair gene regulated by p53, confers resistance to alkylating agents	0.023
*O*-6-methylguanine-DNA methyltransferase		
		
*LIG1* rs3730849 (IVS2+12 C>T) DNA ligase I	LIG1 encodes DNA ligase I, with functions in DNA replication and the base excision repair process. Mutations in LIG1 that lead to DNA ligase I deficiency result in immunodeficiency and increased sensitivity to DNA-damaging agents.	0.031

SNPs with *P*<0.01 (in bold) were selected for further analysis. ^*^P is the overall log-rank *P*-value. Rs numbers and functions are derived from the NCBI Entrez SNP database, accessed December 2008 (http://www.ncbi.nlm.nih.gov/sites/entrez).

**Table 2:** Genotype distributions of SNPs, selected by univariate analysis of PFS, OS and toxicity

**SNP**	**Wild type, *N* (%)**	**Heterozygotes, *N* (%)**	**Homozygote mutants, *N* (%)**	**Total, *N***
*ATM* rs1801516	63 (69.2)	24 (26.4)	4 (4.4)	91
*ERCC5* rs1047768	28 (30.8)	46 (50.5)	17 (18.7)	91
*GADD45A* rs532446	49 (55.1)	31 (34.8)	9 (10.1)	89
*LIG4* rs1805388	62 (68.1)	27 (29.7)	2 (2.2)	91
*BARD1* rs2070093	62 (68.1)	25 (27.5)	4 (4.4)	91
*ERCC2* rs238406	27 (30.0)	46 (51.1)	17 (18.9)	90

**Table 3:** Step II: hazard ratios (CIs) of treatment outcome for colorectal cancer patients receiving oxaliplatin, after adjusting for covariates

**Outcome measure**	**Polymorphism**	**Heterozygote *vs* wild type**	**Homozygote mutant *vs* wild type**	**Carrier analysis**	**Age**	**Performance status**	**Overall *P*^#^**
Progression-free survival	*ATM* rs1801516	0.72 (0.43; 1.21)	4.25 (1.45; 12.44)	4.60 (1.58; 3.37)	0.98 (0.96; 1.01)	1.80 (0.71; 4.55)	**0.009**
			***P*=0.008**	*P*=0.005*			
	*ERCC5* rs1047768	1.71 (0.98; 2.98)	2.85 (1.42; 5.71)	1.92 (1.13; 3.27)	0.98 (0.96; 1.00)	1.62 (0.63; 4.13)	0.012
			***P*=0.003**	*P*=0.016^**^			
	*GADD45A* rs532446	1.58 (0.95; 2.62)	1.14 (0.53; 2.46)	1.44 (1.04; 1.99)	0.98 (0.96; 1.00)	1.56 (0.60; 4.04)	0.216
				*P*=0.029^**^			
Overall survival	*LIG4* rs1805388	—	—	1.83 (1.11; 3.00)	0.98 (0.96; 1.01)	1.42 (0.50; 4.07)	—
				*P*=0.017^**^			
	*BARD1* rs2070093	1.81 (1.05; 3.12)	1.56 (0.56; 4.38)	1.76 (1.05; 2.95)	0.98 (0.96; 1.01)	1.38 (0.44; 3.50)	0.094
		*P*=0.034		*P*=0.031^**^			
Toxicity grade ⩾3	*ERCC2* rs238406	0.36 (0.12; 1.09)	0.10 (0.02; 0.62)	0.28 (0.10; 0.81)	3.07 (0.39; 24.29)	1.09 (1.02; 1.17)	0.028
		*P*=0.070	*P*=0.013	*P*=0.018^**^		***P*=0.008**	

^#^*P* overall log rank *P*-value; carrier analysis: ^*^P wild-type and heterozygotes *vs* homozygote mutants; ^**^P heterozygotes plus homozygote mutants *vs* wild-type patients; performance status defined as stated in the Materials and methods section. All *P*-values in bold are significant according to the threshold set out in the Materials and methods section; other *P*-values are shown for clarification of confidence intervals only. In the carrier analysis, the most appropriate model (recessive or dominant) was chosen for combined analysis of genotypes, based on the individual HR of each category. Owing to the small number of LIG4 rs1805388 homozygote mutant patients, no log rank *P*-value could be calculated to compare the 3 genotypes. SNPs with *P*<0.01 were selected for further analysis.
